# Meiotic Pairing and Segregation of Achiasmate Sex Chromosomes in Eutherian Mammals: The Role of SYCP3 Protein

**DOI:** 10.1371/journal.pgen.0030198

**Published:** 2007-11-02

**Authors:** Roberto de la Fuente, María Teresa Parra, Alberto Viera, Adela Calvente, Rocío Gómez, José Ángel Suja, Julio S Rufas, Jesús Page

**Affiliations:** Unidad de Biología Celular, Departamento de Biología, Universidad Autónoma de Madrid, Madrid, Spain; Stowers Institute for Medical Research, United States of America

## Abstract

In most eutherian mammals, sex chromosomes synapse and recombine during male meiosis in a small region called pseudoautosomal region. However in some species sex chromosomes do not synapse, and how these chromosomes manage to ensure their proper segregation is under discussion. Here we present a study of the meiotic structure and behavior of sex chromosomes in one of these species, the Mongolian gerbil (Meriones unguiculatus). We have analyzed the location of synaptonemal complex (SC) proteins SYCP1 and SYCP3, as well as three proteins involved in the process of meiotic recombination (RAD51, MLH1, and γ-H2AX). Our results show that although X and Y chromosomes are associated at pachytene and form a sex body, their axial elements (AEs) do not contact, and they never assemble a SC central element. Furthermore, MLH1 is not detected on the AEs of the sex chromosomes, indicating the absence of reciprocal recombination. At diplotene the organization of sex chromosomes changes strikingly, their AEs associate end to end, and SYCP3 forms an intricate network that occupies the Y chromosome and the distal region of the X chromosome long arm. Both the association of sex chromosomes and the SYCP3 structure are maintained until metaphase I. In anaphase I sex chromosomes migrate to opposite poles, but SYCP3 filaments connecting both chromosomes are observed. Hence, one can assume that SYCP3 modifications detected from diplotene onwards are correlated with the maintenance of sex chromosome association. These results demonstrate that some components of the SC may participate in the segregation of achiasmate sex chromosomes in eutherian mammals.

## Author Summary

Meiosis is a special kind of cell division that leads to the formation of gametes. During meiosis the number of chromosomes must be halved in the daughter cells, and to do this properly, most organisms use an amazing strategy: during the first of the two meiotic divisions, homologous chromosomes associate in pairs, undergo a reciprocal genetic interchange, and then each member of the pair segregates into a different daughter cell. Genetic exchange, called meiotic recombination, is a key process to ensure that homologous chromosomes remain tightly associated until they segregate. In general, sex chromosomes are subjected to the same processes as the rest of chromosomes. But, of course, exceptions exist. This is the case in the Mongolian gerbil, a mammal whose sex chromosomes pair and segregate during male meiosis without undergoing meiotic recombination. We have found that they are able to do this because some proteins of a meiosis-specific structure, the synaptonemal complex, are reorganized to maintain sex chromosomes associated until they segregate into daughter cells. This kind of behavior resembles the situation found in marsupials and some insect species, indicating a recurrent role of synaptonemal complex components in chromosome segregation when meiotic recombination is missing.

## Introduction

The proper distribution of chromosomes into daughter cells during meiosis depends on the essential phenomena of pairing, synapsis, recombination, and segregation. During early prophase I homologous chromosomes associate in pairs and are held by a proteinaceous structure, the synaptonemal complex (SC) [1–4]. Moreover, homologous chromosomes undergo a process of reciprocal recombination whose cytological manifestation is chiasmata. Recombination between homologues along with the existence of mechanisms that maintain sister chromatid cohesion are responsible for ensuring the proper segregation of homologous chromosomes during first meiotic division [5,6].

It is currently known that these phenomena are intimately related and that they occur in an ordered fashion. Thus, homologous recognition, pairing, and synapsis are promoted by the initiation of recombination events involved in the repair of programmed DNA double strand breaks (DSBs) made by SPO11 protein at the very beginning of first meiotic prophase [7–9]. Furthermore, the assembly of the SC is necessary for the correct completion of recombination and the formation of crossovers [3,10,11]. On the other hand, at least one chiasma per bivalent is necessary to ensure that homologues remain associated from the disorganization of the SC at diplotene until they segregate at anaphase I.

Although this plan is followed by a great majority of species, there are some groups of organisms that show variations in the sequence or even the occurrence of the meiotic hallmarks (for review see [12]). Thus, synapsis precedes recombination initiation in flies and nematodes [13,14]; SC is not formed in dipteran males and fission yeast [15,16]; recombination does not occur in *Drosophila* males and lepidopteran females [17,18]; and in most hemipterans sex chromosome segregation is postponed to the second meiotic division [19,20].

Sex chromosomes are especially prone to get out of the rules of meiosis [21]. In most mammals, sex chromosomes only share a small region of homology named pseudoautosomal region (PAR) [22,23], to which synapsis and recombination are restricted. The occurrence of recombination in the PAR allows sex chromosomes to remain associated until they segregate at anaphase I. However, there are some mammalian species in which the X and Y chromosomes do not form SC. This situation is especially well characterized in marsupials [24–28], in which we have recently reported that a particular structure formed by SC proteins, called dense plate, is involved in maintaining the association of the X and Y chromosomes from pachytene until they segregate at anaphase I [29]. The lack of synapsis has also been reported in some species of eutherian mammals, especially among gerbils and voles [30–34]. In these species sex chromosomes do not form SC, but they are associated during first meiotic prophase and segregate properly during first meiotic division. It has been proposed that in the absence of synapsis, the association of sex chromosomes could be maintained by telomeric or distal heterochromatic associations [30,33,34]. Nevertheless, the nature of the mechanisms that promote sex chromosome pairing and segregation in these species remains unclear.

To shed light on these mechanisms, we have investigated the sequence and the nature of X and Y chromosome association during male meiosis in the Mongolian gerbil (M. unguiculatus), an eutherian mammal that presents asynaptic sex chromosomes [31]. For this purpose we have analyzed the location of SYCP1 and SYCP3 proteins of the SC [35–37], as well as RAD51 and MLH1 proteins, which are involved in meiotic recombination [38,39], and γ-H2AX, a histone variant related to both DSBs′ repair and meiotic sex chromosome inactivation [40,41]. Our results show that even though sex chromosomes in M. unguiculatus neither synapse nor recombine, they pair and remain associated until anaphase I. We have observed structural modifications in their axial elements (AEs) that involve SYCP3 protein, which could be responsible for maintaining sex chromosome association. Since similar results have been reported in marsupials [29], one can assume that the SC plays a crucial and ancient role in the segregation of achiasmate chromosomes.

## Results

### Sex Chromosomes Associate during Prophase I but Do Not Form SC

**Figure 1:**
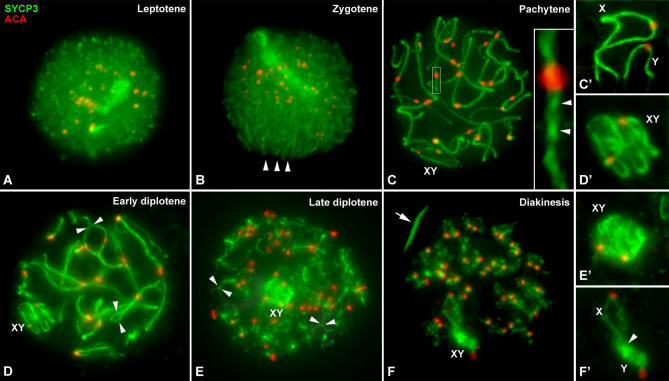
Immunolabeling of Squashed Spermatocytes with Anti-SYCP3 (Green) and Anti-Centromere (Red) Antibodies Several focal planes have been superimposed and projected in a single plane in each image. Selected sex chromosomes from the whole spermatocyte are detailed in the right column at the same meiotic stage of those of the figures. (A) Leptotene: AEs are not completely formed, and they are visualized as thin and discrete lines dispersed in the nucleus. (B) Zygotene: AEs start to form thicker filaments at the regions where synapsis is proceeding. The bouquet configuration is detected by the presence of polarized AE ends in the nucleus (arrowheads). (C) Pachytene: The autosomes have completed their synapsis. Autosomal LEs present helicoidal twists all along their length (arrowheads in the inset). The AEs of the sex chromosomes appear together and are located in the periphery of the nucleus. (C′) Shown is detail of a pachytene spermatocyte in which sex chromosomes are arranged in a front view and their AEs are discernible. Note that there is no contact between them. (D) Early diplotene: SC begins to disorganize, and the LEs appear separated (arrowheads). (D′) The AEs of the sex chromosomes become tangled. (E) Late diplotene: SC has almost completely disassembled from the autosomes, only some portions of the LEs are still synapsed (arrowheads). (E′) SYCP3 on the XY pair redistributes, modifying the AEs morphology, and it begins to accumulate on the Y chromosome. (F) Diakinesis: Autosomes appear compacted with SYCP3 as discontinuous lines along each chromosome. SYCP3 forms aggregates in the cytoplasm as thick bars (arrow). (F′) Sex chromosomes can be distinguished from each other. They appear to be distally connected (arrowhead), and SYCP3 is massively accumulated on the Y chromosome, while on the X it appears as an irregular line along the chromosome.

We first studied the location of SYCP3 protein, the main component of the AE and lateral elements (LEs) of the SC [35,36], on squashed spermatocytes (Figure 1). At leptotene, the signal of SYCP3 is detected as short filaments dispersed in the nucleus (Figure 1A). During zygotene these filaments, corresponding to the AEs, begin to associate in pairs to form thicker filaments (Figure 1B). The typical ”bouquet” arrangement of telomeres is only seen at early zygotene (Video S1), and it usually does not include all the telomeric ends. At pachytene autosomes are associated all along their length (Figure 1C; Video S2). The trajectories of their LEs are clearly discerned, and several twists along each bivalent are detected (Figure 1C, inset). During diplotene, LEs separate (Figure 1D; Video S3), and the SYCP3 signal on the desynapsed LEs becomes thinner at the end of this stage (Figure 1E). At diakinesis SYCP3 is still associated to chromosomes as a discontinuous array of speckles that occupy the region between sister chromatids (Figure 1F). SYCP3 also forms aggregates and irregular bars in the cytoplasm from this stage until the end of first meiotic division.

Sex chromosomal AEs are not distinguishable from that of the autosomes during leptotene (Figure 1A) or zygotene (Figure 1B). The location and morphology of sex chromosomal AEs become evident just at pachytene. At this stage, sex chromosomes are located at the nuclear periphery and occupy a particular domain—the sex body, which presents a higher degree of chromatin condensation compared to the autosomes (unpublished data). The AEs of both X and Y chromosomes are distinguishable one adjacent to the other and inside the sex body. However, they are not in contact, either laterally or distally (Figure 1C and 1C′; Video S4), and they do not show any kind of modifications like thickenings or excrescences, as it is usually found in other mammals [23]. The position of the centromeres along sex chromosomal AEs reveals that the X chromosome is submetacentric and the Y chromosome is metacentric.

During diplotene sex chromosomes remain associated and located at the nuclear periphery. However, as sex chromosomes increase their condensation their AEs fold (Figure 1D and 1D′). At late diplotene, sex chromosome axes become tangled, and the SYCP3 signal shows an intricate morphology making it difficult to discern each sex chromosome inside the sex body (Figure 1E and 1E′). At diakinesis, X and Y chromosomes are distinguishable (Figure 1F and 1F′), and SYCP3 labeling spreads throughout the Y chromosome, while it forms an irregular line running all along the X chromosome. Moreover, sex chromosomes are in contact by an end-to-end association (Figure 1F′). This conformation is maintained until metaphase I.

**Figure 2:**
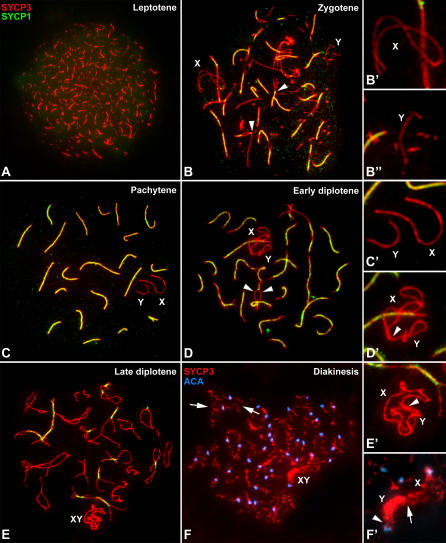
Immunolabeling of Spread Spermatocytes with Anti-SYCP3 (Red) and Anti-SYCP1 (Green) Antibodies (A–E) and Anti-SYCP3 (Red) and Anti-Centromere (Blue) Antibodies (F) (A) Leptotene: SYCP3 is detected as short lines dispersed in the whole nucleus. No signal of SYCP1 is detected. (B) Zygotene: SYCP3 is detected over the autosomal AEs, which appear partially synapsed (arrowheads). SYCP1 is detected in the regions that have already synapsed. Synapsis proceeds from different points along chromosomes. Sex chromosome AEs also appear labeled with anti-SYCP3 but no SCYP1 labeling is detected (detailed in B′–B′′). X and Y chromosomes appear separated in the nucleus. (C) Pachytene: SYCP3 and SYCP1 labeling on the autosomes are coincident. Sex chromosomes (enlarged in C′) appear in a close position, but their AEs do not contact and they do not show SYCP1 labeling. (D) At early diplotene, SYCP1 begins to dissociate from the SC, and the LEs can be seen separated in certain regions along the bivalents (arrowheads). Sex chromosomal AEs appear folded and distally connected. One end of the AE of the Y chromosome (see detail in D′) is in contact with both tips of the X chromosome (arrowhead). (E) Late diplotene. The bivalents show very little SYCP1 signal. Sex chromosomal AEs are tangled, and their outline is fairly irregular (as seen in E′). The four chromosomal ends seem to be clustered in a single point (arrowhead in E′). (F) Diakinesis: SYCP3 over the autosomes is detected as a zigzagging and curly signal. Some chiasmata points are clearly identifiable (arrows). (F′) The sex chromosomes appear to be distally connected (arrow). SYCP3 labeling on the X chromosome is similar to the labeling on the autosomes, while the labeling is massive over the Y chromosome, excepting in the pericentromeric region (arrowhead).

To test the asynaptic nature of the sex chromosome association in *M. unguiculatus,* we carried out the double immunolocalization of SYCP3 and SC central element protein SYCP1 [35,37] on spermatocyte spreads (Figure 2). SYCP1 is not detected at leptotene (Figure 2A), while at zygotene short stretches of signals appear between the AEs of homologous chromosomes located either at distal or interstitial regions (Figure 2B). Interestingly, SYCP1 association to the chromosomes starts before all AEs are completely formed. At pachytene, synapsis is completed in the autosomal bivalents, and the signals of SYCP1 and SYCP3 are coincident along the bivalents (Figure 2C). At diplotene, SYCP1 dissociates from the bivalents and the LEs begin to separate (Figure 2D and 2E).

Contrary to what was observed in squashes, sex chromosome AEs can be identified on spreads during zygotene (Figure 2B and 2B′). This is most probably due to the higher degree of chromatin dispersion produced with this technique. At this stage sex chromosomal AEs are completely formed, and their thickness is similar to that of the autosomal AEs. The AEs of X and Y chromosomes can be found either closely located or separated in the nucleus at mid zygotene, and even at late zygotene sex chromosomes still remain separated in 41.7% of the cells (*n* = 60) (Figure S1). Nevertheless, from early pachytene onwards they are always closely related (Figure 2C). Two features indicate that the assembly of the SC central element is not involved in this association: first, sex chromosomal AEs do not show any physical contact; and second, SYCP1 protein is completely absent from the X and Y chromosomes (Figure 2C′). However, the ends of sex chromosome AEs are distally connected at the beginning of diplotene (Figure 2D and 2D′). This contact may involve any of the ends of each sex chromosome with the other sex chromosome or even the two tips of each chromosome, as shown for sex chromosomes of other species of *Gerbillidae* [30,31,33]. Nevertheless, at late diplotene the four chromosome ends are always connected (Figure 2E and 2E′). Noticeably, no SYCP1 signal is found in these regions of distal association.

As observed in squashed preparations, the morphology of sex chromosomal AEs is modified from diplotene onwards when studied on spreads. Thus, AEs become irregular and folded at diplotene (Figure 2D and 2E′), and at diakinesis SYCP3 expands to form a massive and intense signal that seems to cover the whole Y chromosome (Figure 2F and 2F′).

### Location of Recombination-Related Proteins in the Sex Chromosomes

**Figure 3:**
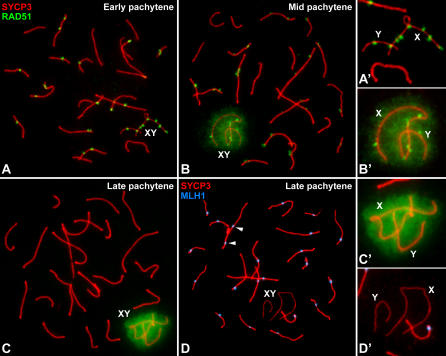
Immunolocalization of SYCP3 (Red) and RAD51 (Green) (A–C) and SYCP3 (Red) and MLH1 (Blue) (D) Proteins on Spread Spermatocytes (A–A′) At early pachytene RAD51 foci are visible over the autosomes. This protein also appears associated to the AEs of sex chromosomes (enlarged in A′). (B–B′) At mid pachytene the number of RAD51 foci over the autosomes and sex chromosomes decreases, but RAD51 begins to accumulate over the sex chromatin as a diffuse signal. (C–C′) At late pachytene RAD51 is absent from autosomes but intensely extended on the sex chromatin. (D) MLH1 protein is detectable at late pachytene as one single dot over most of the autosomal axes, although some bivalents present two (arrowheads). No signal of this protein is detected over the axes of the sex chromosomes (D′).

In mammals the initiation of SC assembly is dependent on the occurrence of previous recombination events [9,41]. Therefore, we wondered whether the absence of synapsis between sex chromosomes could be due to the absence of such recombination events in them. We analyzed the location of RAD51, a protein related to early repair of DSBs, and MLH1 protein, which is related to the last steps of recombination leading to the formation of crossovers (Figure 3) [42,43].

RAD51 is detected on the autosomal AEs during zygotene and early pachytene as dots on or very close to the AEs/LEs (Figure 3A). The number of RAD51 dots decreases at mid pachytene (Figure 3B), and it is absent from the autosomes at late pachytene (Figure 3C). Sex chromosomes, particularly the X chromosome, also exhibit RAD51, as several dots on the AEs (Figure 3A and 3B′). At mid pachytene (Figure 3B and 3B′), the number of dots on the AEs decreases and is undetectable at late pachytene (Figure 3C and 3C′). It is interesting to note the persistence of many RAD51 foci on the sex chromosomes at mid pachytene, while most of RAD51 foci have disappeared from autosomes (Figure 3B). Additionally, the detection of a faint RAD51 labeling on the sex chromatin at early-mid pachytene is also intriguing (Figure 3B′). This labeling persists and becomes even more intense in some late pachytene spermatocytes (Figure 3C′).

MLH1 is only detected at late pachytene on autosomal SCs. Most bivalents present one MLH1 focus, but some of them may present two foci (Figure 3D). However, MLH1 is not detected on the sex chromosomes (Figure 3D and 3D′).

### SYCP3 Appears Connecting Sex Chromosomes at Latter Stages of First Meiotic Division

**Figure 4:**
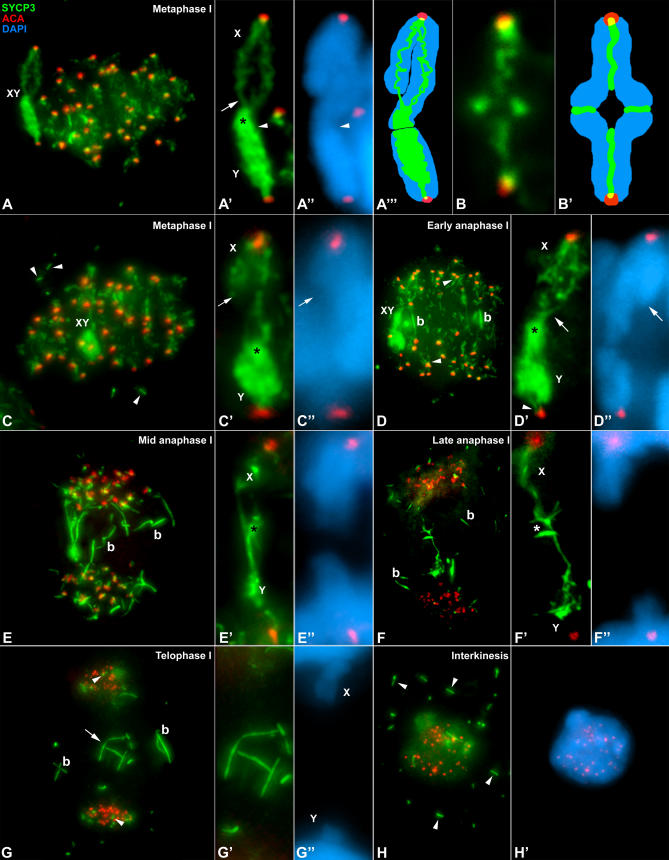
Immunolabeling of Squashed Spermatocytes with Anti-SYCP3 (Green) and Anti-Centromere (Red) Antibodies Several focal planes have been superimposed and projected in each image. (A) Metaphase I. Bivalents are correctly bioriented in the metaphase plate, including the XY pair. (A′–A′′) SYCP3 is detected on the X chromosome as an irregular line (with small splittings and excrescences) covering the interchromatid domain. The Y chromosome is completely labeled with the anti-SYCP3 antibody. Comparison of SYCP3 and DAPI images shows that this massive labeling also involves the distal region of the X chromosome long arm (asterisk in A′–A′′). The distal region of the long arm of the X chromosome contacts with the Y chromosome (arrowhead) while an SYCP3 filament overpasses the X short arm and links to the distal region of the X long arm and the Y chromosome (arrow). (A′′′) Schematic illustration of the XY pair in this stage. The limit between both sex chromosomes is marked. (B) Shown is an autosomal bivalent in metaphase I, and (B′) its schematic representation. SYCP3 signal runs along the interchromatid domain and interrupts at the chiasma point. (C–C′′) Metaphase I: In this spermatocyte bivalents are correctly bioriented in the metaphase plate, including the XY pair. Some aggregates of SYCP3 are detected in the cytoplasm (arrowheads). In this case, the SYCP3 bridge from the X chromosome short arm is broken (arrow in C′ and C′′), while the long arm is still in contact with the Y chromosome (asterisk). (D–D′′) Early anaphase I: SYCP3 begins to dissociate from the autosomes as they migrate to the poles, but some SYCP3 is still present near some centromeres (arrowheads) and along some regions of the chromosome arms. Bar-shaped SYCP3 aggregates appear in the spindle area of the cytoplasm (b). Although sex chromosomes initiate their migration they remain linked by an SYCP3 bridge. Note that in this case the filament protruding from the X short arm is still visible (arrow in D′). SYCP3 signal exceeds the end of the X chromosome short arm (arrow in D′′). SYCP3 is detected as a filament in the pericentromeric region of the Y chromosome (arrowhead in D′), in contrast with the massive signal observed on it. The SYCP3 massive labeling has partially disorganized but it does not disappear (asterisk). During mid (E–E′′) and late anaphase I (F–F′′), this massive SYCP3 joining between X and Y chromosomes disorganizes, making it difficult to unequivocally identify sex chromosomes as they move apart from each other (their putative location has been indicated as X and Y). A series of thick filaments are present between chromatin masses migrating to opposite poles (asterisk). (G–G′′) At telophase I thick SYCP3 filaments are visible in the cytoplasm between the cell poles (arrow) and also around some centromere regions (arrowheads). (H) Interkinesis. Minute bars of SYCP3 are still detected in the cytoplasm (arrowheads).

Given the striking modification of SYCP3 location during late stages of prophase I, we analyzed SYCP3 distribution during late stages of first meiotic division to ascertain its potential role in sex chromosome segregation (Figure 4).

At metaphase I, SYCP3 protein remains associated with autosomes at the region of sister chromatid contact (the interchromatid domain) (Figure 4A and 4B). This pattern of localization is similar to that described in mouse and other mammals and can be related to a role for SYCP3 in maintaining sister chromatid cohesion [44,45]. Nevertheless, this protein does not accumulate in the centromeric regions, as occurs in mouse [44].

The pattern of SYCP3 localization on the X chromosome is visualized as a sinuous and irregular line that runs along its interchromatid domain (Figure 4A and 4A′′). In contrast, SYCP3 signal on the Y chromosome is not restricted to the interchromatid domain, but occupies almost the entire width of the chromatin, excepting the pericentromeric region, in which the protein is present only as a thin filament (Figures 4A′, 4C′, 4D′, and S2). As described above, this pattern of SYCP3 distribution appears during diakinesis and is subsequently maintained in later stages. However, once sex chromosomes are pulled to the spindle poles at metaphase I, it becomes evident that the extensive labeling of SYCP3 involves not only the Y chromosome but also the most distal segment of the X chromosome long arm (Figures 4A′, 4C′, 4D′, and S2).

At metaphase I, sex chromosomes are associated and properly bioriented. However, we observed two different configurations. In the first configuration, both arms of the X chromosome are in contact with the Y chromosome (Figure 4A), and there is a clear continuity between SYCP3 signals on the X and Y chromosomes. Interestingly, SYCP3 signal may overpass the length of the short arm of the X chromosome and contact with the massive SYCP3 labeling that covers the distal region of the X long arm and the Y chromosome (Figure 4A′ and A′′′). In the second configuration, the bridge of SYCP3 signal breaks in the short arm of the X chromosome, but remains intact between the end of the X long arm and the Y chromosome (Figure 4C and 4C′′; Video S5). Therefore, the association of both chromosomes seems to be maintained by SYCP3 and not just by a direct contact of the chromatin of the X and Y chromosomes.

At the beginning of anaphase I, SYCP3 dissociates from the chromosomes but does not disappear abruptly since it is still detectable during early anaphase I at the interchromatid domains, mainly close to the centromeres (Figure 4D). The SYCP3 bridge between X and Y chromosomes also persists in anaphase I. As observed in metaphase I, at the beginning of anaphase I SYCP3 filaments may appear either connecting both X and Y chromosomal ends (Figure 4D and 4D′′; Video S6) or just the long arm of the X chromosome with either one or both of the Y chromosome arms (unpublished data). Later in anaphase I, SYCP3 disappears from the interchromatid domain of autosomes and is only detectable in some pericentromeric regions (Figure 4E and 4E′′). SYCP3 is also visible as filaments located inside and outside the spindle area that are not in contact with the chromosomes. Nevertheless, one of these filaments is usually detected connecting the chromosomes of opposite anaphase I poles (Figure 4F and 4F′′). SYCP3 is still detected at telophase I as filaments present near the centromeric regions of chromosomes and as longer filaments dispersed over the protoplasm (Figure 4G). Some of these filaments are visible during interkinesis (Figure 4H), while no SYCP3 labeling is detectable during the second meiotic division (unpublished data).

**Figure 5:**
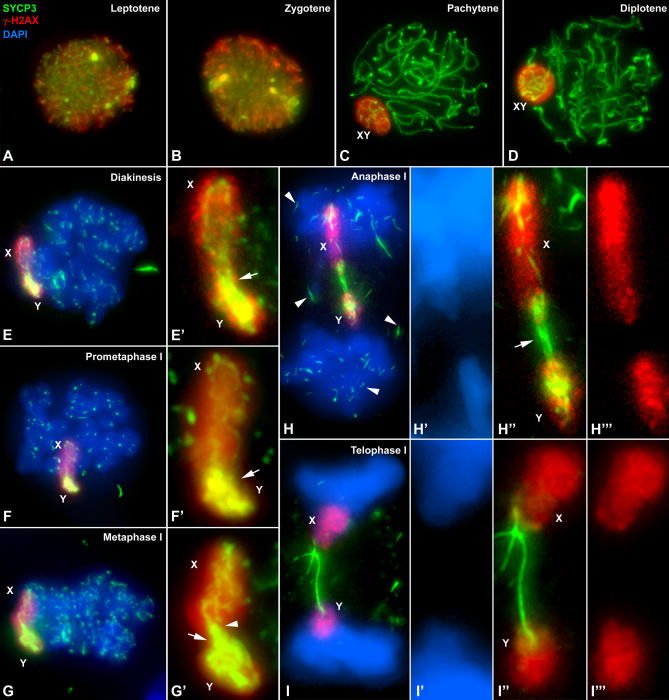
Immunolabeling of Squashed Spermatocytes with Anti-SYCP3 (Green) and Anti γ-H2AX (Red) Antibodies and Counterstaining of Chromatin with DAPI (Blue) Several focal planes have been projected in each image. (A) Leptotene: γ-H2AX labeling appears distributed throughout the whole nucleus. (B) During zygotene γ-H2AX distribution is very similar to that observed in the previous stage. (C) From pachytene onwards, the bulk of anti-γ-H2AX antibody is located onto the sex chromosomes, which appear located at the nuclear periphery and remain so during diplotene (D). (E–E′) Diakinesis: Sex chromosomes appear distally connected and intensely labeled with γ-H2AX. Note the SYCP3 connection between both arms of the sex chromosomes (arrow in E′). The same situation is found in prometaphase I (F–F′). (G–G′) Metaphase I: The autosomal bivalents remain aligned at the metaphase I plate. The connection between the sex chromosomes has broken in the X chromosome short arm (arrowhead), and only the long arm is linked to the Y (arrow). (H–H′′′) Anaphase I: Sex chromosomes migrate to the poles, but they are delayed compared to autosomes. Note the remnants of SYCP3 over the autosomes and dispersed in the cytoplasm (arrowheads). A thick SYCP3 filament is visible between the X and Y chromosomes (arrow), but no chromatin joining is detected as revealed by DAPI (H′) or γ-H2AX staining (H′′′). (I–I′′′) Telophase I: Sex chromosomes are clearly lagged in segregation. The SYCP3 filament appears connecting them without chromatin connection (I′–I′′′).

Taking into account the pattern of SYCP3 distribution on the sex chromosomes up to metaphase I, it is likely that some of the SYCP3 filaments found during anaphase I associate the sex chromosomes. With the aim of identifying the X and Y chromosomes inside these chromatin masses and their relation to SYCP3 filaments, we carried out the double immunolabeling of SYCP3 and γ-H2AX (Figure 5), a phosphorylated form of histone variant H2AX. In mouse, γ-H2AX decorates the entire nucleus during leptotene and early zygotene in response to DSBs and is thereafter restricted to the chromatin of the sex body from late zygotene until diplotene, where it is related to the process of meiotic sex chromosome inactivation [40,41]. We found that in M. unguiculatus γ-H2AX occupies the whole nucleus at leptotene (Figure 5A) and zygotene (Figure 5B). From pachytene onwards γ-H2AX is almost exclusively located on the sex chromosomes. However, contrary to what occurs in mouse, it does not disappear at diplotene but remains detectable until telophase I (Figure 5C–5I). Thus, the signal of the γ-H2AX allowed us to unequivocally identify the sex chromosomes during the late stages of the first meiotic division.

The simultaneous labeling of SYCP3 and γ-H2AX corroborates that SYCP3 occupies almost the entire width of the Y chromosome during diakinesis up to metaphase I (Figure 5E–5G′; Video S7). At the beginning of anaphase I, the massive SYCP3 signals found on the Y chromosome and the distal region of the X chromosome disorganize and the sex chromosomes migrate to opposite poles (Figure 5H). Moreover, the SYCP3 filament detected during anaphase I actually bridges the sex chromosomes (Figure 5H and 5H′′′; Video S8). Additionally, we observed that the sex chromosomes are always lagged during anaphase I migration. However, this feature seems not to be due to a chromatin association of the sex chromosomes since no chromatin bridges are detected either with DAPI (Figure 5H′ and 5I′) or γ-H2AX staining (Figure 5H′′′ and 5I′′′).

## Discussion

One of the most striking advances in the understanding of meiosis in the past years has been the realization that the particular processes that take place during this special kind of cell division are tightly interrelated [11]. The interdependence of pairing, synapsis, recombination and segregation has been demonstrated in a series of model species, including yeast, mammals, and plants. However, the characterization of species that present deviations from this paradigm is especially valuable to understand the universality of these rules. In particular, the study of species in which synapsis and recombination are absent is relevant to discover alternative mechanisms that can promote chromosomes to properly recognize, associate, and segregate during meiosis [29].

### Lack of PAR Is Responsible for the Asynaptic Nature of Sex Chromosomes in M. unguiculatus

Our analysis of the sequence of SC assembly in the Mongolian gerbil revealed that both X and Y chromosome assemble an AE, but they do not synapse. A first explanation for this behavior is that the mechanisms that promote chromosome synapsis in mammals, i.e., occurrence and repair of DNA DSBs [9,41], do not take place on the sex chromosomes of M. unguiculatus. It has been demonstrated that the disruption of DNA DSB occurrence and/or repair severely impairs synapsis in mouse [9,46,47]. Moreover, it has been reported that in the grasshopper Stethophyma grossum large portions of the autosomes remain unsynapsed during first meiotic prophase due to the absence of DNA DSBs [48]. However, our results on the location of RAD51 in the sex chromosomes of M. unguiculatus suggest that asynapsis is not due to the absence DSB recombinational repair.

A second explanation is that sex chromosomes in the Mongolian gerbil do not share a region of homology. Thus, although sex chromosomes can initiate the processes that ultimately culminate in the synapsis with the homologous chromosome, they are unable to complete this process because they have no homologous partner. In this sense, the absence of synapsis between sex chromosomes appears to be a recurrent feature among the species of the family *Gerbillidae*. This is the case of Psamommys obessus [30,33], Gerbillus campestris, *M. libycus, M. shawi*, and M. crassus [32]. On the other hand, some species present sex chromosomes with synapsis and recombination, as in G. chiesmani, *G. nigeriae*, *G. hoogstrali*, and Taterillus pygargus [31]. However, the synapsing regions in these species seem to be originated, as in many other eutherian mammals, by recent translocations of autosomal segments to both the X and Y chromosomes [49,50]. Previous analyses on the sex chromosomal phylogeny of *Gerbillidae* have shown that the X chromosome of M. unguiculatus could be one of the most primitive among this family [49], reinforcing the idea that the asynaptic condition of sex chromosomes would be an ancient feature of this group.

Therefore, the absence of PAR is to us the most plausible explanation for the absence of synapsis between the X and the Y chromosomes. However, it has been reported that in some species, the marsupial Macropus eugenii for instance, sex chromosomes do not synapse even though they share a region of homology [51]. In the same way, our current knowledge of the human X and Y chromosomes reveals that they still share many segments with different degrees of homology that lay out of the regions usually involved in the formation of SC [52,53]. Therefore, in the absence of direct DNA sequence comparison it is not possible to rule out the possibility that some homology is still shared between sex chromosomes in M. unguiculatus. Nonetheless, these homologous regions could be degenerated or reorganized in such a way that they would not be able to promote synapsis any longer, i.e., they would lack a sort of ”functional homology”.

### Maintenance of Sex Chromosome Association at Pachytene Does Not Depend on AE's Distal Associations

Our data indicate that pairing of sex chromosomes takes place during zygotene, and they remain associated at pachytene. However, the lack of the PAR between sex chromosomes in M. unguiculatus poses an interesting question about the mechanisms that could be involved in bringing and maintaining them together during the first meiotic prophase.

As regards the first topic, one could assume that the polarization of telomeres during the bouquet stage plays an important role in the initial approach of sex chromosomes. Nevertheless, this mechanism would not be sufficient to ensure sex chromosome pairing, since they can appear close together at the very beginning of zygotene, before autosomal AEs are completely formed, and on the contrary, they can remain separated in the nucleus until late zygotene, well after the resolution of the bouquet.

Another possibility, although highly speculative, is that sex chromosome pairing is based on a mechanism of homologous sequence recognition. As mentioned above, the absence of a functional PAR does not imply that there is not homology at all between sex chromosomes. Provided that a certain degree of homology could be conserved, it is possible that the mechanisms of DNA repair mediated by RAD51 and other proteins could promote the approaching and recognition of X and Y chromosomes, although, as stressed before, structural or genetic factors would hamper the formation of a SC. In this sense, this residual homology could not be as efficient as a PAR in promoting the recognition of sex chromosomes, explaining their erratic behavior during zygotene.

Once sex chromosomes recognize each other they remain intimately paired throughout pachytene, even though SC is not formed. In other *Gerbillidae* species, sex chromosomes present some kind of distal connections between the ends of their AEs at pachytene [30,32,33]. These associations may be autologous or heterologous, and it has been claimed that they would be ultimately responsible for ensuring sex chromosome association [30]. However, this may not be the case for sex chromosomes in M. unguiculatus since X and Y chromosomes do not present distal contacts of their AEs at pachytene, a stage that lasts several days in the Mongolian gerbil [54]. Distal connections between the tips of the AEs are only found from early diplotene onwards. Therefore, other mechanisms must be discussed. Nevertheless, it is possible that all the combinations of distal association previously reported [30,32,33] may respond to a sequential and random clustering of telomeres, which culminates in the association of all AE tips at late pachytene/early diplotene, and that the differences found between species are simply due to different timing in the association of sex chromosomal ends.

An alternative explanation is that the particular chromatin condensation of the sex body may contribute to maintain the association of X and Y chromosomes. It is currently known that sex chromosomes are transcriptionally inactive during most of the first meiotic prophase in mammals and a huge number of proteins, including γ-H2AX, are specifically associated to and/or modified in the sex body [41,55,56]. In this sense, it has been demonstrated that disruption of H2AX in mouse abolishes meiotic sex chromosome inactivation and sex body formation [40]. Furthermore, in H2AX-depleted mice sex chromosome pairing is severely disturbed and X and Y chromosomes are often located separately in the nucleus. Our results on the location of γ-H2AX indicate that sex chromosomes in the Mongolian gerbil are inactivated and form a compacted chromatin mass at the nuclear periphery. Therefore, it is possible that the chromatin conformation acquired during first meiotic prophase could have an important role in maintaining sex chromosome association in absence of SC formation.

### SYCP3 Is Involved in Maintaining the Association of the Sex Chromosomes up to Anaphase I and in Ensuring Their Correct Segregation

**Figure 6:**
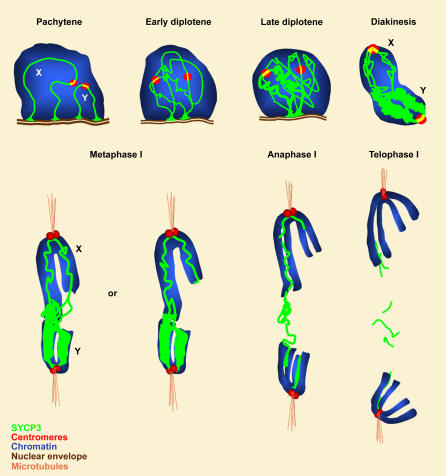
Schematic Representation of the Pairing and Segregation Dynamics of the Sex Chromosomes in M. unguiculatus Male Meiosis The AEs of the sex chromosomes appear physically separated at pachytene, but closely related at the periphery of the nucleus. As the chromosomes increase their condensation at early diplotene, the AEs tend to fold and their tips establish an end-to-end contact. At the end of diplotene, SYCP3 reorganizes on the AEs, and acquires a diffuse pattern, mainly on the Y chromosome. At diakinesis sex chromosomes are end-to-end connected, in a conformation that is maintained up to metaphase I, and SYCP3 appears covering the Y chromosome and the distal region of the X chromosome. Our proposal is that the physical connection mediated by SYCP3 at this stage is responsible for maintaining sex chromosome association. Once sex chromosomes have achieved a bipolar orientation on the metaphase I spindle they tend to move to opposite poles. At this point the SYCP3-mediated association may break at the short arm of the X chromosome. At anaphase I chromosomes move to opposite poles. However, an SYCP3 filament remains associating both sex chromosomes, probably causing the delay of sex chromosome migration at anaphase I. This filament ultimately detaches from sex chromosomes during telophase I.

The correct segregation of chromosomes during first meiotic division depends on their proper alignment and biorientation at the metaphase I plate. In M. unguiculatus, as occurs in other *Gerbillidae* species, X and Y chromosomes appear distally associated at metaphase I. However, the absence of a chiasma between sex chromosomes, as revealed by the absence of MLH1, poses an intriguing question about the mechanism that maintains their association. In P. obessus, this association is mediated by distal blocks of heterochromatin [30]. However, our results in the Mongolian gerbil reveal this distal association may not be mediated just by chromatin interactions. We suggest that the physical connection mediated by SYCP3 protein, starting at the latest stages of the first meiotic prophase, may be responsible for maintaining sex chromosomes connected (Figure 6). This SYCP3 link would prevent X and Y chromosomes to separate each other before they biorientate at the metaphase I plate. Afterward, as soon as each chromosome is pulled to the spindle poles, sex chromosomes would tend to separate displaying an early segregation. This would explain our finding of different configurations in the association of sex chromosomes at metaphase I.

Since sex chromosomes always appear as laggards at anaphase I it seems that their movement to the poles is somehow obstructed. Physical links between segregating half bivalents at anaphase I have been detected in a wide range of species [57] but are specially well characterized in crane flies [58]. In these species, the presence of elastic tethers between the ends of segregating chromosomes has been reported, giving rise to the stretch of the chromosome arms [59]. The nature of such bridges remains obscure, but it was proposed that they could be formed by chromatin fibers [58] or even by elements related to the attachment of telomeres to the nuclear envelope [59]. In this way, it is likely that the SYCP3 filaments that connect sex chromosomes in M. unguiculatus, by establishing a physical link between their ends, could act also as a tether that retards sex chromosome separation. However, we do not favor the idea that chromatin could be also involved in this connection because we were unable to detect any link by either DAPI or γ-H2AX labeling.

The critical feature in this context is how the AEs components derive in such a structure. Previous studies have shown that the elements of the SC can be transformed into a variety of structures that may remain associated to chromosomes until anaphase I [60,61]. Furthermore, in vitro experiments have shown that both SYCP3 and SYCP1 are able to self assemble into filaments and polycomplex-like structures, respectively [62,63]. Our observations indicate that in the Mongolian gerbil SYCP3 reorganization initiates at diplotene, concomitant with the initiation of sex chromosome end-to-end connections. An electron-dense irregular network has been detected at late pachytene on the sex chromosomes in other gerbil species, soon after the establishment of sex chromosome distal associations [31,32]. Therefore, it is likely that other *Gerbillidae* species also present a similar SYCP3 reorganization. Although the timing of these changes may differ between species, they could be a part of a conserved program of sex chromosome modification at the late stages of first meiotic prophase. Interestingly, in marsupial mammals a structure derived from the sex chromosomal AEs, the dense plate, is also involved in both pairing [24] and segregation [29] of the achiasmate sex chromosomes. Although differences exist between the organization and behavior of the SYCP3 structures characterized in marsupials and in the Mongolian gerbil, these findings indicate an evolutionarily conserved role of SC components not only in synapsis but also in chromosome segregation when synapsis and/or recombination do not take place.

Finally, the finding that SYCP3 may form conspicuous aggregates in the cytoplasm, which is a feature common to other species of mammals, is also remarkable [44]. In the Mongolian gerbil these structures, mainly bars and filaments, are detected from diakinesis up to interkinesis. Our results suggest that the origin of these structures may be multiple: (i) aggregates formed at later stages of the first meiotic prophase (diakinesis), and which remain out of the nucleus until interkinesis; (ii) filaments in the spindle area that could derive directly from the SYCP3 on the autosomes at the beginning of anaphase I; and (iii) filaments that presumably dissociate from the SYCP3 link between the X and Y chromosomes during anaphase I and telophase I. The formation of these filaments could be related to the tendency of SYCP3 to form filaments when over expressed in vitro [62].

Different animal groups challenge the rule that synapsis and recombination are required for proper segregation. It is well known that insects represent a wide range of segregation mechanisms of achiasmate chromosomes [12,17,20,64]. The observations presented here and those of previous authors indicate that, at least in mammals, special chromatin conformations (heterochromatinization and/or inactivation) and modification of SC components may be key mechanisms to explain the proper association and segregation of achiasmate chromosomes [29,30,33,34,65]. Since the occurrence of such chromosomes is a feature found in almost all groups of organisms, they may represent universal backup mechanisms to ensure the correct outcome of meiosis in the absence of synapsis and recombination.

## Materials and Methods

Testes of adult M. unguiculatus (*Gerbillidae*) males were extracted and dissected in PBS (137 mM NaCl, 2.7 mM KCl, 10.1 mM Na_2_HPO_4_, and 1.7 mM KH_2_PO_4_ [pH 7.4]) to obtain the seminiferous tubules, which were subsequently processed for either squashing or spreading techniques. Squash was carried out as described by Page et al. [66]. Briefly, tubules were fixed for 10 min in 2% formaldehyde in PBS containing 0.05% Triton X-100 (Sigma) and then several pieces were minced with tweezers, placed on a slide coated with 1 mg/ml poly-L-lysine (Sigma), and subsequently squashed. Slides were then frozen in liquid nitrogen and immediately immersed in PBS after removing the coverslip. Spreading techniques were performed as described by Peters et al. [67]. Seminiferous tubules were disaggregated in PBS, and the cell suspension centrifuged at 1,200 rpm for 8 min and cells resuspended in PBS, centrifuged again, and resuspended in 100 mM sucrose. Cells were then simultaneously spread onto a slide and fixed with formaldehyde 1% in distilled water containing 50 mM Na_2_B_4_O_7_ and 0.15% Triton X-100. After air drying, slides were washed with 0.04% Photo-Flo (Kodak) in distilled water and air dried before used for immunofluorescence.

### Immunofluorescence.

Slides were incubated overnight at 4 °C with the following primary antibodies diluted in PBS: mouse monoclonal anti-SYCP3 (Abcam, 12452) at a 1:100 dilution; rabbit anti-SYCP3 (Abcam, 15093) at a 1:50 PBS dilution; rabbit anti-SYCP1 (Abcam, 15087) at a 1:100 dilution; mouse monoclonal against histone H2AX phosphorylated at serine 139 (γ-H2AX) (Upstate, 05–636) at a 1:3,000 dilution; rabbit anti-RAD51 (Calbiochem, PC130) at a 1:50 dilution; mouse monoclonal anti-MLH1 (Pharmingen, 551091 ) at a 1:10 dilution; and a human anti-centromere serum that recognizes centromeric proteins (Antibodies Incorporated, 15–235) at a 1:100 dilution. Slides were rinsed 3 × 5 min in PBS and subsequently incubated with secondary antibodies in a moist chamber at room temperature for 1 h: fluorescein isothiocyanate (FITC)-conjugated goat anti-mouse IgG; Texas Red (TR)-conjugated goat anti-mouse IgG; FITC-conjugated goat anti-rabbit IgG; TR-conjugated goat anti-rabbit IgG; and TR-conjugated goat anti-human IgG. All secondary antibodies were from Jackson (Jackson ImmunoResearch Laboratories) and used a 1:100 dilution. Slides were subsequently rinsed in PBS 3 × 5 minutes, stained with DAPI, and mounted with Vectashield (Vector). For double detection of two antibodies raised in mouse, we followed the procedure previously described [24].

Observations were made on an Olympus BX61 microscope equipped with a motorized Z axis. Images were captured with an Olympus DP70 digital camera using the analySIS software (Soft Imaging System, Olympus) and processed by using public domain ImageJ (National Institutes of Health, http://rsb.info.nih.gov/ij) and Adobe Photoshop 7.0 software.

## Supporting Information

Figure S1 Immunolabeling of SYCP3 (Green) in Two Late Zygotene Spermatocytes in Which Sex Chromosomes Are Separated (A) and Closely Located (B) In both cases a late-synapsing autosomal bivalent can be observed (asterisk). We scored sex chromosomes as separated when the distance between their AEs is longer than the length of the X chromosome AE. Frequencies of both situations are detailed below the images.
(2.0 MB TIF)

Figure S2 Double Immunolocalization of SYCP3 (Green) and ACA (Red) and Counterstaining of Chromatin with DAPI (Blue) in a Metaphase I Spermatocyte Only some images from the 3-D reconstruction are projected to observe the sex chromosomes.
(A) Note the signal of SYCP3 as a thin filament in the pericentromeric region of the Y chromosome (arrow). The aggregate of SYCP3 (asterisk) in the long arm of the X chromosome connects it with the Y chromosome (arrowhead).
(B) The chromatin of both chromosomes is not in contact (arrowhead), and it is clear that the aggregate of SYCP3 relies on the distal segment of the X chromosome.
(1.2 MB TIF)

Video S1 Zygotene This video, as well as Video S4, corresponds to the 3-D reconstructions of the cells represented in Figure 1B and 1C′, respectively. Double immunolocalization of SYCP3 (green) and ACA (red). In this video, the telomere clustering region is marked (arrow).
(2.1 MB MOV)

Video S2 Pachytene Double immunolocalization of SYCP3 (green) and ACA (red). Shown is 3-D reconstruction of a pachytene spermatocyte in which the XY pair is labeled on the lower part of the movie.
(2.5 MB MOV)

Video S3 Diplotene Double immunolocalization of SYCP3 (green) and ACA (red). SC begins to dissociate from the autosomes and their LEs are detected separated. The XY pair is labeled on the right part of the image.
(2.1 MB MOV)

Video S4 Detail of the XY Pair in a Pachytene Nucleus Double immunolocalization of SYCP3 (green) and ACA (red). By reconstructing the nucleus, X and Y chromosomes can clearly be seen not in contact with each other.
(1.1 MB MOV)

Video S5 X and Y Chromosomes at the Metaphase I Plate Double immunolocalization of SYCP3 (green) and ACA (red) and staining with DAPI (blue). The sex chromosomes are arranged in the metaphase I plate (labeled), and the short arm of the X chromosome has dissociated from the Y. The 3-D reconstruction allows us to distinguish the chromatin of the short arm separated from the chromatin of the Y chromosome.
(8.9 MB MOV)

Video S6 Early Anaphase I This video corresponds to the 3-D reconstruction of the cell in Figure 4D. Double immunolocalization of SYCP3 (green) and ACA (red). SYCP3 is still joining the distal part of the X chromosome (X) with the Y chromosome (Y), and some filaments of the protein are detached from the autosomes.
(1.9 MB MOV)

Video S7 Metaphase I This video, as well as Video S8, corresponds to the 3-D reconstruction of the spermatocytes in Figure 5G and 5H, respectively. Double immunolocalization of SYCP3 (green) and γ-H2AX (red). The signal of γ-H2AX unequivocally identifies the sex chromosomes (labeled), in addition with the massive signal of SYCP3. The long arm of the X chromosome is still in contact with the Y chromosome.
(1.1 MB MOV)

Video S8 Anaphase I Double immunolocalization of SYCP3 (green) and γ-H2AX (red). The X and Y chromosomes are labeled in the image, identified by the γ-H2AX signal. They are clearly linked by a SYCP3 aggregate.
(1.4 MB MOV)

## Abbreviations

AE: axial element
DSB: double strand break
LE: lateral element
PAR: pseudoautosomal region
SC: synaptonemal complex

## References

[pgen-0030198-b001] Fawcett DW (1956). The fine structure of chromosomes in the meiotic prophase of vertebrate spermatocytes. J Biophys Biochem Cytol.

[pgen-0030198-b002] Moses MJ (1956). Chromosomal structures in crayfish spermatocytes. J Biophys Biochem Cytol.

[pgen-0030198-b003] Page SL, Hawley RS (2004). The genetics and molecular biology of the synaptonemal complex. Annu Rev Cell Dev Biol.

[pgen-0030198-b004] Heyting C (1996). Synaptonemal complexes: structure and function. Curr Opin Cell Biol.

[pgen-0030198-b005] Miyazaki WY, Orr-Weaver TL (1994). Sister-chromatid cohesion in mitosis and meiosis. Annu Rev Genet.

[pgen-0030198-b006] Suja JA, Antonio C, Rufas JS (1992). Involvement of chromatid cohesiveness at the centromere and chromosome arms in meiotic chromosome segregation: a cytological approach. Chromosoma.

[pgen-0030198-b007] Grelon M, Vezon D, Gendrot G, Pelletier G (2001). AtSPO11–1 is necessary for efficient meiotic recombination in plants. Embo J.

[pgen-0030198-b008] Keeney S, Giroux CN, Kleckner N (1997). Meiosis-specific DNA double-strand breaks are catalyzed by Spo11, a member of a widely conserved protein family. Cell.

[pgen-0030198-b009] Romanienko PJ, Camerini-Otero RD (2000). The mouse Spo11 gene is required for meiotic chromosome synapsis. Mol Cell.

[pgen-0030198-b010] de Vries FA, de Boer E, van den Bosch M, Baarends WM, Ooms M (2005). Mouse Sycp1 functions in synaptonemal complex assembly, meiotic recombination, and XY body formation. Genes Dev.

[pgen-0030198-b011] Zickler D, Kleckner N (1999). Meiotic chromosomes: integrating structure and function. Annu Rev Genet.

[pgen-0030198-b012] Wolf KW (1994). How meiotic cells deal with non-exchange chromosomes. Bioessays.

[pgen-0030198-b013] Dernburg AF, McDonald K, Moulder G, Barstead R, Dresser M (1998). Meiotic recombination in C. elegans initiates by a conserved mechanism and is dispensable for homologous chromosome synapsis. Cell.

[pgen-0030198-b014] McKim KS, Green-Marroquin BL, Sekelsky JJ, Chin G, Steinberg C (1998). Meiotic synapsis in the absence of recombination. Science.

[pgen-0030198-b015] Olson L, Eden U, Egel-Mitani M, Egel R (1978). Asynaptic meiosis in fission yeast?. Hereditas.

[pgen-0030198-b016] Rasmussen SW (1973). Ultrastructural studies of spermatogenesis in Drosophila melanogaster Meigen. Z Zellforsch Mikrosk Anat.

[pgen-0030198-b017] Rasmussen SW (1977). The transformation of the Synaptonemal Complex into the ‘elimination chromatin' in Bombyx mori oocytes. Chromosoma.

[pgen-0030198-b018] Morgan TH (1912). Complete linkage in the second chromosome of the male of Drosophila. Science.

[pgen-0030198-b019] González-García JM, Antonio C, Suja JA, Rufas JS (1996). Meiosis in holocentric chromosomes: kinetic activity is randomly restricted to the chromatid ends of sex univalents in Graphosoma italicum (Heteroptera). Chromosome Res.

[pgen-0030198-b020] Ueshima N (1979). Animal cytogenetics. Insecta 6. Hemiptera: Heteroptera.

[pgen-0030198-b021] Page J, de la Fuente R, Gomez R, Calvente A, Viera A (2006). Sex chromosomes, synapsis, and cohesins: a complex affair. Chromosoma.

[pgen-0030198-b022] Burgoyne PS (1982). Genetic homology and crossing over in the X and Y chromosomes of mammals. Hum Genet.

[pgen-0030198-b023] Solari AJ (1974). The behavior of the XY pair in mammals. Int Rev Cytol.

[pgen-0030198-b024] Page J, Berrios S, Rufas JS, Parra MT, Suja JA (2003). The pairing of X and Y chromosomes during meiotic prophase in the marsupial species Thylamys elegans is maintained by a dense plate developed from their axial elements. J Cell Sci.

[pgen-0030198-b025] Roche L, Seluja G, Wettstein R (1986). The meiotic behaviour of the XY pair in Lutreolina crassicaudata (Marsupialia: Didelphoidea). Genetica.

[pgen-0030198-b026] Seluja G, Roche L, Solari AJ (1987). Male meiotic prophase in Didelphis albiventris. J Heredity.

[pgen-0030198-b027] Sharp P (1982). Sex chromosome pairing during male meiosis in marsupials. Chromosoma.

[pgen-0030198-b028] Solari AJ, Bianchi NO (1975). The synaptic behaviour of the X and Y chromosomes in the marsupial Monodelphis dimidiata. Chromosoma.

[pgen-0030198-b029] Page J, Viera A, Parra MT, de la Fuente R, Suja JA (2006). Involvement of synaptonemal complex proteins in sex chromosome segregation during marsupial male meiosis. PLoS Genet.

[pgen-0030198-b030] Ashley T, Moses MJ (1980). End association and segregation of the achiasmatic X and Y chromosomes of the sand rat, Psammomys obesus. Chromosoma.

[pgen-0030198-b031] Ratomponirina C, Viegas-Pequignot E, Dutrillaux B, Petter F, Rumpler Y (1986). Synaptonemal complexes in Gerbillidae: probable role of intercalated heterochromatin in gonosome-autosome translocations. Cytogenet Cell Genet.

[pgen-0030198-b032] Ratomponirina C, Viegas-Pequignot E, Petter F, Dutrillaux B, Rumpler Y (1989). Synaptonemal complex study in some species of Gerbillidae without heterochromatin interposition. Cytogenet Cell Genet.

[pgen-0030198-b033] Solari AJ, Ashley T (1977). Ultrastructure and behavior of the achiasmatic, telosynaptic XY pair of the sand rat (Psammomys obesus). Chromosoma.

[pgen-0030198-b034] Wolf KW, Baumgart K, Winking H (1988). Meiotic association and segregation of the giant sex chromosomes in male field vole (Microtus agrestis). Chromosoma.

[pgen-0030198-b035] Dobson MJ, Pearlman RE, Karaiskakis A, Spyropoulos B, Moens PB (1994). Synaptonemal complex proteins: occurrence, epitope mapping and chromosome disjunction. J Cell Sci.

[pgen-0030198-b036] Lammers JHM, Offenberg HH, van Aalderen M, Vink ACG, Dietrich AJJ (1994). The gene encoding a major component of the lateral elements of synaptonemal complexes of the rat is related to X-linked lymphocyte-regulated genes. Mol Cell Biol.

[pgen-0030198-b037] Meuwissen RLJ, Offenberg HH, Dietrich AJJ, Riesewijk A, van Iersel M (1992). A coiled-coil related protein specific for synapsed regions of meiotic prophase chromosomes. Embo J.

[pgen-0030198-b038] Ashley T, Plug AW, Xu J, Solari AJ, Reddy G (1995). Dynamic changes in Rad51 distribution on chromatin during meiosis in male and female vertebrates. Chromosoma.

[pgen-0030198-b039] Baker SM, Plug AW, Prolla TA, Bronner CE, Harris AC (1996). Involvement of mouse Mlh1 in DNA mismatch repair and meiotic crossing over. Nat Genet.

[pgen-0030198-b040] Fernandez-Capetillo O, Mahadevaiah SK, Celeste A, Romanienko PJ, Camerini-Otero RD (2003). H2AX is required for chromatin remodeling and inactivation of sex chromosomes in male mouse meiosis. Dev Cell.

[pgen-0030198-b041] Mahadevaiah SK, Turner JM, Baudat F, Rogakou EP, de Boer P (2001). Recombinational DNA double-strand breaks in mice precede synapsis. Nat Genet.

[pgen-0030198-b042] Marcon E, Moens PB (2005). The evolution of meiosis: recruitment and modification of somatic DNA-repair proteins. Bioessays.

[pgen-0030198-b043] Plug AW, Peters AH, Keegan KS, Hoekstra MF, de Boer P (1998). Changes in protein composition of meiotic nodules during mammalian meiosis. J Cell Sci.

[pgen-0030198-b044] Parra MT, Viera A, Gomez R, Page J, Benavente R (2004). Involvement of the cohesin Rad21 and SCP3 in monopolar attachment of sister kinetochores during mouse meiosis I. J Cell Sci.

[pgen-0030198-b045] Moens PB, Spyropoulos B (1995). Immunocytology of chiasmata and chromosomal disjunction at mouse meiosis. Chromosoma.

[pgen-0030198-b046] Barchi M, Mahadevaiah S, Di Giacomo M, Baudat F, de Rooij DG (2005). Surveillance of different recombination defects in mouse spermatocytes yields distinct responses despite elimination at an identical developmental stage. Mol Cell Biol.

[pgen-0030198-b047] Liebe B, Petukhova G, Barchi M, Bellani M, Braselmann H (2006). Mutations that affect meiosis in male mice influence the dynamics of the mid-preleptotene and bouquet stages. Exp Cell Res.

[pgen-0030198-b048] Calvente A, Viera A, Page J, Parra MT, Gomez R (2005). DNA double-strand breaks and homology search: inferences from a species with incomplete pairing and synapsis. J Cell Sci.

[pgen-0030198-b049] Viegas-Pequignot E, Benazzou T, Dutrillaux B, Petter F (1982). Complex evolution of sex chromosomes in Gerbillidae (Rodentia). Cytogenet Cell Genet.

[pgen-0030198-b050] Graves JA, Wakefield MJ, Toder R (1998). The origin and evolution of the pseudoautosomal regions of human sex chromosomes. Hum Mol Genet.

[pgen-0030198-b051] Toder R, Wienberg J, Voullaire L, O'Brien PC, Maccarone P (1997). Shared DNA sequences between the X and Y chromosomes in the tammar wallaby - evidence for independent additions to eutherian and marsupial sex chromosomes. Chromosoma.

[pgen-0030198-b052] Rozen S, Skaletsky H, Marszalek JD, Minx PJ, Cordum HS (2003). Abundant gene conversion between arms of palindromes in human and ape Y chromosomes. Nature.

[pgen-0030198-b053] Skaletsky H, Kuroda-Kawaguchi T, Minx PJ, Cordum HS, Hillier L (2003). The male-specific region of the human Y chromosome is a mosaic of discrete sequence classes. Nature.

[pgen-0030198-b054] Segatelli TM, Almeida CC, Pinheiro PF, Martinez M, Padovani CR (2002). Kinetics of spermatogenesis in the Mongolian gerbil (Meriones unguiculatus). Tissue Cell.

[pgen-0030198-b055] Hoyer-Fender S (2003). Molecular aspects of XY body formation. Cytogenet Genome Res.

[pgen-0030198-b056] McKee BD, Handel MA (1993). Sex chromosomes, recombination, and chromatin conformation. Chromosoma.

[pgen-0030198-b057] Suja JA, Antonio C, Debec A, Rufas JS (1999). Phosphorylated proteins are involved in sister-chromatid arm cohesion during meiosis I. J Cell Sci.

[pgen-0030198-b058] Fuge H (1978). Fine structure of anaphase bridges in meiotic chromosomes of the crane fly Pales. Chromosoma.

[pgen-0030198-b059] LaFountain JR, Cole RW, Rieder CL (2002). Partner telomeres during anaphase in crane-fly spermatocytes are connected by an elastic tether that exerts a backward force and resists poleward motion. J Cell Sci.

[pgen-0030198-b060] Moens PB, Church K (1979). Distribution of synaptonemal complex material in metaphase-I bivalents of Locusta and Chloealtis (Orthoptera, Acrididae). Chromosoma.

[pgen-0030198-b061] Esponda P, Stockert JC (1972). Evolution of synaptonemal complex in helix-aspersa spermatocytes. Chromosoma.

[pgen-0030198-b062] Yuan L, Pelttari J, Brundell E, Bjorkroth B, Zhao J (1998). The synaptonemal complex protein SCP3 can form multistranded, cross-striated fibers in vivo. J Cell Biol.

[pgen-0030198-b063] Ollinger R, Alsheimer M, Benavente R (2005). Mammalian protein SCP1 forms synaptonemal complex-like structures in the absence of meiotic chromosomes. Mol Biol Cell.

[pgen-0030198-b064] Wahrman J, Nezer R, Freund O (1973). Multiple chromosome mechanisms with ‘segregation bodies'. Chromosomes Today.

[pgen-0030198-b065] Page J, Berrios S, Parra MT, Viera A, Suja JA (2005). The program of sex chromosome pairing in meiosis is highly conserved across marsupial species: implications for sex chromosome evolution. Genetics.

[pgen-0030198-b066] Page J, Suja JA, Santos JL, Rufas JS (1998). Squash procedure for protein immunolocalization in meiotic cells. Chromosome Res.

[pgen-0030198-b067] Peters AH, Plug AW, van Vugt MJ, de Boer P (1997). A drying-down technique for the spreading of mammalian meiocytes from the male and female germline. Chromosome Res.

